# Execution by Electricity

**Published:** 1895-11

**Authors:** 


### EXECUTION BY ELECTRICITY.

Not very long since, M. D'Arsonval, of Paris, asserted that in the attempt at Electrocution in the New York prisons, the life of the criminal was taken, not by the electro-executioner, but by the medical attendants at the "autopsy." Such a statement, from such an authority, furnished the newspapers with some choice sensational matter which was rapidly and thoroughly worked up.

To investigate the matter, Governor Flower appointed Dr. A. H. Goelet and Mr. A. E. Kennelly, a practical electrician, to witness the execution of the negro Hampton, January 28, 1895. These gentlemen reported that the execution was conducted as usual, a current pressure of 1,740 volts being used. The electrical charge which the body received was alternately increased and diminished during the 57 seconds continuation of the current. Then the "heart had ceased to beat, the pupils were dilated, there was slight froth about the mouth, the face was pale and the skin of the upper thorax showed a marked redness or hyperemia which disappeared on pressure but slowly returned. The moment the current was turned on, the whole body was thrown into intense tetanic rigidity, there was some frothing at the mouth but no sound was made. Death was instantaneous and painless." At the autopsy it was seen that "all the blood in the body had collected in the tissues of the scalp and brain, and in the abdominal organs. The rest of the body was bloodless. Many of the blood-vessels of the head were ruptured, and a large quantity of pure blood was found in the dura mater, and heavy clots were found at the base of the brain. The authors conclude that while in cases of accidental contact with live wires resuscitation should always be tried because of imperfection of contact, yet, when a current of 8 *ampères* at 1,740 volts is deliberately passed through the body, death is instantaneous, painless and absolute. The disorganization of the brain-tissues and blood-vessels is alone sufficient to cause immediate death without the slightest possibility of resuscitation."

We note that Judge J. W. Goff, of the New York police court, is a strong advocate of electrocution. He says that it is the most humane form of inflicting death, and that all countries will eventually adopt it. It is a step in the direction of progressive civilization.

